# Truncating Variants in *RFC1* in Cerebellar Ataxia, Neuropathy, and Vestibular Areflexia Syndrome

**DOI:** 10.1212/WNL.0000000000201486

**Published:** 2023-01-31

**Authors:** Riccardo Ronco, Cecilia Perini, Riccardo Currò, Natalia Dominik, Stefano Facchini, Alice Gennari, Roberto Simone, Skye Stuart, Sara Nagy, Elisa Vegezzi, Ilaria Quartesan, Amar El-Saddig, Timothy Lavin, Arianna Tucci, Agnieszka Szymura, Luiz Eduardo Novis De Farias, Alexander Gary, Megan Delfeld, Priscilla Kandikatla, Nifang Niu, Sanjukta Tawde, Joseph Shaw, James Polke, Mary M. Reilly, Nick W. Wood, Emmanuele Crespan, Christopher Gomez, Jin Yun Helen Chen, Jeremy Dan Schmahmann, David Gosal, Henry Houlden, Soma Das, Andrea Cortese

**Affiliations:** From the Department of Neuromuscular Diseases (R.R., R.C., N.D., S.F., Alice Gennari, R.S., S.S., S.N., A.T., A.S., L.E.N.D.F., M.M.R., N.W.W., H.H., A.C.), UCL Queen Square Institute of Neurology, London, United Kingdom; Department of Brain and Behavioral Sciences (R.R., R.C., I.Q., A.C.), University of Pavia, Pavia, Italy; Institute of Molecular Genetics IGM-CNR “Luigi Luca Cavalli-Sforza” (C.P., E.C.), Italy; Department of Neurology (S.N.), University Hospital Basel, University of Basel, Switzerland; IRCCS Mondino Foundation (E.V.), Pavia, Italy; Manchester Centre for Clinical Neurosciences (A.E.-S., T.L., D.G.), Salford Royal Hospital, Northern Care Alliance NHS Foundation Trust, Manchester, United Kingdom; Clinical Pharmacology (A.T.), William Harvey Research Institute, School of Medicine and Dentistry, Queen Mary University of London, United Kingdom; Departamento de Distúrbios do Movimento (L.E.N.D.F.), Hospital Das Clínicas Da Universidade Federal Do Paraná, Curitiba, Brazil; University of Chicago Medical Center (Alexander Gary, M.D., P.K., S.D.), The University of Chicago, IL; Department of Human Genetics (N.N., S.T.), The University of Chicago, IL; Neurogenetics (J.S., J.P.), University College London Hospitals NHS Foundation Trust, National Hospital for Neurology and Neurosurgery, London, United Kingdom; Department of Neurology (C.G.), The University of Chicago, IL; and Ataxia Center (J.Y.H.C., J.D.S.), Laboratory for Neuroanatomy and Cerebellar Neurobiology, Department of Neurology, Massachusetts General Hospital and Harvard Medical School, Boston.

## Abstract

**Background and Objective:**

Cerebellar ataxia, neuropathy, and vestibular areflexia syndrome (CANVAS) is an autosomal recessive neurodegenerative disease characterized by adult-onset and slowly progressive sensory neuropathy, cerebellar dysfunction, and vestibular impairment. In most cases, the disease is caused by biallelic (AAGGG)_n_ repeat expansions in the second intron of the replication factor complex subunit 1 (*RFC1*). However, a small number of cases with typical CANVAS do not carry the common biallelic repeat expansion. The objective of this study was to expand the genotypic spectrum of CANVAS by identifying sequence variants in *RFC1*-coding region associated with this condition.

**Methods:**

Fifteen individuals diagnosed with CANVAS and carrying only 1 heterozygous (AAGGG)_n_ expansion in *RFC1* underwent whole-genome sequencing or whole-exome sequencing to test for the presence of a second variant in *RFC1* or other unrelated gene. To assess the effect of truncating variants on *RFC1* expression, we tested the level of RFC1 transcript and protein on patients' derived cell lines.

**Results:**

We identified 7 patients from 5 unrelated families with clinically defined CANVAS carrying a heterozygous (AAGGG)_n_ expansion together with a second truncating variant *in trans* in *RFC1*, which included the following: c.1267C>T (p.Arg423Ter), c.1739_1740del (p.Lys580SerfsTer9), c.2191del (p.Gly731GlufsTer6), and c.2876del (p.Pro959GlnfsTer24). Patient fibroblasts containing the c.1267C>T (p.Arg423Ter) or c.2876del (p.Pro959GlnfsTer24) variants demonstrated nonsense-mediated mRNA decay and reduced RFC1 transcript and protein.

**Discussion:**

Our report expands the genotype spectrum of RFC1 disease. Full *RFC1* sequencing is recommended in cases affected by typical CANVAS and carrying monoallelic (AAGGG)_n_ expansions. In addition, it sheds further light on the pathogenesis of RFC1 CANVAS because it supports the existence of a loss-of-function mechanism underlying this complex neurodegenerative condition.

Cerebellar ataxia, neuropathy, and vestibular areflexia syndrome (CANVAS) is an autosomal recessive neurodegenerative disease characterized by adult-onset and slowly progressive ataxia caused by the contemporary impairment of sensory neurons, the vestibular system, and the cerebellum.^1-3^ In most cases, the disease is caused by biallelic (AAGGG)_n_ repeat expansion in the second intron of the replication factor complex subunit 1 (*RFC1*) gene.^4-21^ *RFC1* encodes the large subunit of replication factor C, a 5-subunit DNA polymerase accessory protein required for the coordinated synthesis of both DNA strands during replication and after DNA damage.

Notably, previous studies in biallelic (AAGGG)_n_ expansion carriers using patients' cell lines and 1 postmortem brain did not show a reduction of RFC1 RNA or protein nor an overt dysfunction of DNA replication and repair.^4,9^ This is puzzling because recessively inherited conditions are typically associated with loss of function of the mutant gene and suggests that more complex mechanisms may be involved in the pathogenesis of RFC1 CANVAS. In this study, we present the first 7 cases from 5 unrelated families affected by the typical CANVAS phenotype carrying a heterozygous (AAGGG)_n_ repeat expansion together with a second truncating variant in *RFC1*.

## Methods

### Patients

DNA samples were collected from patients diagnosed with ataxia, sensory neuropathy, or CANVAS at the National Hospital for Neurology and Neurosurgery at Queen Square Institute of Neurology (London, United Kingdom), at the University of Chicago Genetic Services Laboratory (Chicago), and internationally through collaborating centers between January 2019 and May 2022. Patients were diagnosed as experiencing CANVAS based on the characteristic combination of sensory neuronopathy, cerebellar dysfunction, and bilateral vestibular areflexia.^1^

### *RFC1* Testing

*RFC1* genetic test was performed using repeat primed PCR and flanking PCR, as previously described.^4,21^ When sufficient DNA was available, Southern blotting was also performed.^4^

### Whole-Genome Sequencing and Whole-Exome Sequencing

Individuals diagnosed with CANVAS and carrying only 1 heterozygous (AAGGG)_n_ expansion in *RFC1* underwent whole-genome sequencing (WGS) or whole-exome sequencing (WES). WGS and WES were performed by Macrogen (Netherlands) or at the University of Chicago. At Macrogen, paired-end sequencing reads (150 bp) were generated using a Novaseq 6000 system (Illumina) and aligned to GRCh38 using the Burrows-Wheeler Aligner.^22^ The mean coverage per sample was 40x. Variants were called according to the Genome Analysis Toolkit HaplotypeCaller workflow^23^ and annotated with Ensembl Variant Effector Predictor.^24^ Variants were prioritized based on segregation, minor allele frequency (<0.0001 in the 1000 Genomes Project,^25^ National Heart, Lung, and Blood Institute [NHLBI] GO Exome Sequencing Project (ESP) (Exome Variant Server, NHLBI GO ESP,^26^ or the Genome Aggregation Database^27^), evolutionary conservation, and in silico prediction of pathogenicity for coding variants. The process of WES at the University of Chicago was similar with the following exceptions: exome sequencing was performed using the IDT xGEN Exome Research kit (Integrated DNA Technologies), and sequence was aligned to GRCh37. Variants were annotated and evaluated using a validated custom bioinformatic pipeline.

### *RFC1* Expression Studies in Patient-Derived Cell Lines

To assess the effect of truncating variants on *RFC1* expression, patients' fibroblasts from affected (I-1 and I-2) and unaffected (I-3) individuals from family 1, case II from family 2, patients carrying biallelic AAGGG expansion (n = 5), and age-matched and sex-matched controls (n = 5) were harvested and grown in culture in Dulbecco's Modified Eagle Media (DMEM) supplemented with 15% fetal bovine serum (Euroclone). Real-time quantitative PCR (RT-qPCR) and Western blotting were replicated on biologically independent samples at least twice with similar results.

### *RFC1* Gene Silencing in HEK293 Cell Lines

In order to validate the specificity of the anti-RFC1-p140 mouse monoclonal antibody (1:2′500, a kind gift of Ulrich Hubscher^28^), we performed *RFC1* gene silencing as described in.^29^ In brief, HEK293 human neuroblastoma from European Collection of Cell Culture (ECACC) were cultured in a T75 flask and maintained in DMEM (Thermo Fisher Scientific, Waltham, MA) supplemented with 10% Fetal Bovine Serum (Thermo Fisher Scientific) at 37°C in a 5% CO_2_ humidified incubator. HEK293 were splitted at ≈80% confluence and replated in a 6-well plate (800.000 cells per well). To silence RFC1, 25 pmol of siRNA pool (siRFC1: 5′-GUAAAUAGCUCCCGUAAAG-3′; 5′-GGAAUUAAUUGGCCUGAUA -3′; 5′-GUCCAAAGAUCUAAUAAGA-3′; 5′-CAUAUGCGAUGGUGACCUA-3′) or nontargeting siRNA (D-001810-10-05, Thermo Fisher scientific) was mixed with 7.5 μL of Lipofectamine RNAiMAX (Invitrogen) following manufacturer's transfection protocol and applied to each well of a 6-well plate. Forty-eight hours later, transfected cells were collected for subsequent analysis.

### Real-Time Quantitative PCR

Total RNA was extracted from fibroblasts using RNAeasy Mini kit (Qiagen, Hilden, Germany) and treated with RNAse-free DNase I (Qiagen). Complementary DNA (cDNA) was synthesized using 500 ng of total RNA for all samples, with a SuperScript III First Strand cDNA Synthesis kit (Invitrogen, Waltham, MA) and an equimolar mixture of oligo (dT)18 and random hexamer primers. Real-time qRT-PCR was perform using the Power SYBR Green Master Mix (Applied Biosystems, Waltham, MA) and measured with a QuantStudio 7 Flex Real-Time PCR System (Applied Biosystems). *RFC1* expression level (forward: 5′-CTTCGCGGGAGAAGTTGTTG-3′, reverse: 5′-ATTTCCGAATGTCCATCGCAG-3′) was normalized to GAPDH (forward = 5′-TGCACCACCAACTGCTTAGC-3′, reverse = 5′-GGCATGGACTGTGGTCATGAG-3') and RPL10 (forward = 5′-AATCTCCAGGGGCACCATT-3′, reverse = CGCTGGCTCCCACTTTGT-3′). Amplified transcripts were quantified using the comparative CT method and presented as normalized fold expression change (2^ΔΔCt^).

To assess the allelic status of the c.2876del (p.Pro959GlnfsTer24) truncating variant mapping to exon 22 in individual II, we took advantage of the presence of a c.2511T>C (p.Ser837=) (rs2066782) synonymous variant on exon 19, which is part of the same haplotype containing the (AAGGG)n repeat expansion. cDNA from patient's fibroblasts was amplified by touchdown PCR encompassing exons 17–23 (forward:5′-TCTTCGTTTTCAAAGACCTCGGG-3′, reverse:5′-TTTGCCACCCCAGCTGCTG-3′), and Sanger sequencing was performed on exon 19 (forward: 5′-TGGGAGCCAATCAAGATATCAGA-3′, reverse: 5′-GCTGCAAACACTTTCCGGGC-3′) and exon 22 (forward: 5′-CTGGAGTCTTCTGCCTGCGC-3′, reverse: 5′-GGTCAAGGGCTGTACAAGTGCA-3′) using both forward and reverse primers. For comparison, PCR and sequencing of exon 19 (forward:5′-GGTGTGCACGAAGTAAAGCA-3′, reverse: 5′-ATTCCACAGGCATACCAAGG-3’) and exon 22 (forward:5′-TGGATCAAGGTGTGTACCGC-3′, reverse: 5′-CCCGAACAGAGTAATCCCAC -3′) of *RFC1* were also performed on gDNA from case II.

### Western Blotting

Cells were collected in cold PBS 1X, resuspended in radioimmunoprecipitation Assay buffer (1% Nonidet P-40, 50 mM Tris-HCl [pH 8.0], 150 mM NaCl, 0.1% SDS, 0.1% DOC) supplemented with 1X protease inhibitor cocktail (Sigma Aldrich, St. Louis, MO) and 1X phosphatase inhibitor cocktail (Roche Diagnostics, Basel, Switzerland), incubated for 20 minutes on ice, then centrifuged (15 minutes at 6′200*g*, 4°C), and the protein-containing supernatant was collected. Protein lysate concentrations were assessed using Protein Assay Dye Reagent Concentrate (Bio-Rad, Hercules, CA). Lysates (25 μg) were mixed with 1X cracking buffer (300 mM Tris-HCl [pH 6.8], 10% SDS, 40% glycerol, 600 mM DTT, 5% 2-mercaptoethanol, and 0.2% bromophenol-blue) and denatured for 5 minutes at 95°C. Samples were separated in 8% SDS-polyacrylamide gel in 1X Tris/glycine/SDS running buffer and transferred onto a nitrocellulose membrane using a Turbo Transfer Pack (Bio-Rad). Membranes were then blocked for 1 hour in 6% skimmed milk and incubated overnight at 4°C with 2.5% skimmed milk with the following antibodies recognizing human proteins: anti-RFC1-p140 mouse monoclonal (1:2′500, a kind gift of Ulrich Hubscher^28^), anti-GAPDH mouse monoclonal (cat. no. ab8245; 1:10,000; Abcam), or anti–α-actinin (cat. No. 3134, Cell Signaling). Specificity of anti-RFC1-p140 was validated by assessing binding in siRFC1-HEK293 cell line. Primary antibodies were probed with horseradish peroxidase–conjugated secondary antibodies (antimouse, cat. no. 115-035-146; 1:5′000; Jackson ImmunoResearch Laboratories, Inc, West Grove, PA) for 1 hour at room temperature. Protein bands were visualized using an enhanced chemiluminescent reagent (Westar Eta C Ultra 2.0; Cyanagen, Bologna BO, Italy). The intensity of the band in each sample was quantified using QuantityOne software (Bio-Rad Laboratories, Inc), corrected for the intensity of the corresponding band obtained with the anti-GAPDH antibody, and normalized to the control sample.

### RNA-seq

Library preparation and RNAseq were performed at UCL genomics. Reads were aligned to the hg38 human genome build using STAR (c 2.7.7a).^30^ BAM files were sorted and duplicate reads flagged using umi_tools (version 1.1.1).^31^ The aligned reads overlapping the human genes (Gencode v35) were counted with featureCounts (version 2.0.1).^32^

### Standard Protocol Approvals, Registrations, and Patient Consents

The study was approved by local institutional ethical committees. Written informed consent was obtained from patients, older than 18 years, participating in the study.

### Data Availability

Anonymized data from this study will be shared by request from any qualified investigator.

## Results

### *RFC1* Genetic Studies

**Figure 1:**
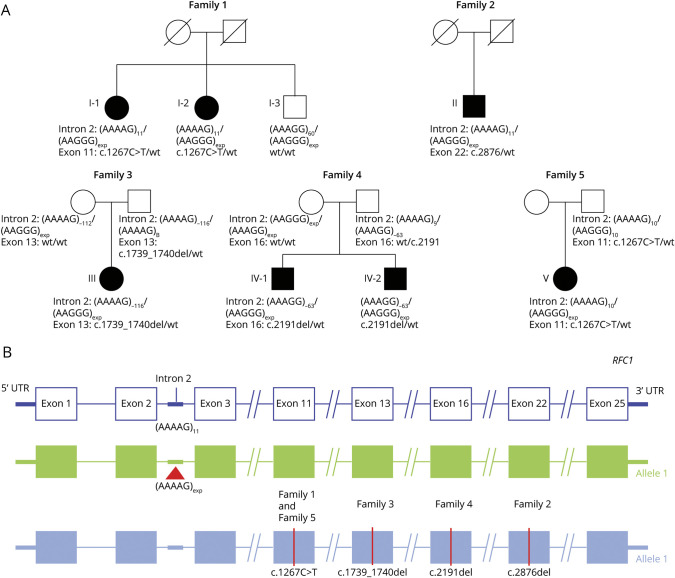
Truncating Variants in *RFC1* in Compound Heterozygous State With Pathogenic (AAGGG)n Expansions Cause CANVAS (A) Family pedigrees. (B) Schematic diagram showing the location of mutations identified in this study on *RFC1* gene (NM_002913.5). Abbreviation: CANVAS = cerebellar ataxia, neuropathy, and vestibular areflexia syndrome; *RFC1* = replication factor complex subunit 1.

As part of *RFC1* genetic testing at the Queen Square Institute of Neurology and at the University of Chicago, we identified 15 patients from 13 families with a clinical diagnosis of typical CANVAS who carried only 1 heterozygous (AAGGG)_n_ expanded allele. WES or WGS was performed and identified a second truncating variant in *RFC1* in 7 of them, from 5 unrelated families (5/13, 38%), who are further described in this study (Figure 1). No other relevant variant, including splicing or noncoding variants in *RFC1* or other genes, was identified in the remaining 7 families.

### Case Description

#### Family 1

I-2 was referred for neurologic evaluation for progressive imbalance, reduced sensation in her hands and feet, and oscillopsia since her mid-50s. She had also complained of chronic cough since the age of 15 years.

Neurologic examination in I-2 at the age of 61 years showed a broad-based gait and positive Romberg. There was gaze-evoked nystagmus, broken pursuits, absent vestibular ocular reflex bilaterally, and altered visually enhanced vestibulo-ocular reflex (VVOR). Sensation to pinprick was reduced all over including the face, while sensation to vibration was reduced to the sternum in the upper limbs and to the anterior superior iliac spine in the lower limbs. Joint position was preserved. Reflexes were reduced in the upper and lower limbs. There was mild dysmetria to finger-nose and heel-shin. Tone, muscle bulk, and power were normal (Video 1).

10.1212/201486_Video_1 Video 1 Download Supplementary Video 1 via http://dx.doi.org/10.1212/201486_Video_1

Nerve conduction studies showed absent sensory action potentials in the upper and lower limbs but normal motor studies. MRI showed vermian atrophy involving the ventral and dorsal lobules and cord parenchymal volume loss that involved the entire cord and preferentially the dorsal aspect, wherein faint longitudinally extensive T2 hyperintensity was shown to involve the dorsal columns. Vestibular testing confirmed the presence of bilateral vestibular failure.

In her family, her older sister (I-1), now aged 62 years, also reported similar symptoms, while their younger brother (I-3) and their parents were unaffected.

I-1 describes the onset of a chronic cough from her early 20s. Since her late 40s, she has been complaining of unsteadiness, worse in the dark, followed a few years later by neuropathic pain and oscillopsia related to head movements, with difficulty reading road signs when walking. The condition progressed and she developed dysarthria, swallowing difficulties, and autonomic involvement with symptomatic orthostatic hypotension. Examination and investigations at the age of 62 years supported a clinical diagnosis of CANVAS.

I-3 did not report any symptoms. He had a normal examination and normal nerve conduction studies at the age of 52 years.

*RFC1* testing in I-1 and I-2 showed the presence of heterozygous (AAGGG)_n_ expansion of 1,000 repeats together with a nonexpanded (AAAAG)_11_ allele. I-3 also carried the (AAGGG)_1000_ expansion in compound heterozygous state with a small nonpathogenic expansion of (AAAGG)_60_ repeats on the other allele. WGS was thus performed and showed in I-1 and I-2, but not in I-3, the presence of a c.1267C>T (p.Arg423Ter) truncating variant in *RFC1* (NM_002913.5).

#### Family 2

Case II is a 64-year-old man who has been complaining since his late 40s of progressive imbalance, which was worse in the dark, associated with numbness, paresthesia, and diffuse neuropathic pain in his 4 limbs coupled with an intense cold pain sensation affecting his torso. Oscillopsia and symptoms suggestive of autonomic failure, including orthostatic hypotension with syncopal episodes and bladder, bowel, and sexual dysfunction, were also later reported. He was adopted and has never had any contact with his biological family.

Examination at the age of 64 years showed bilateral vestibular areflexia, together with signs of cerebellar dysfunction and sensory impairment. Brain MRI showed mild cerebellar atrophy. Nerve conduction studies revealed the presence of a severe sensory neuronopathy with absent sensory action potentials throughout but normal motor studies. Vestibular testing showed bilateral vestibular areflexia.

**Figure 2:**
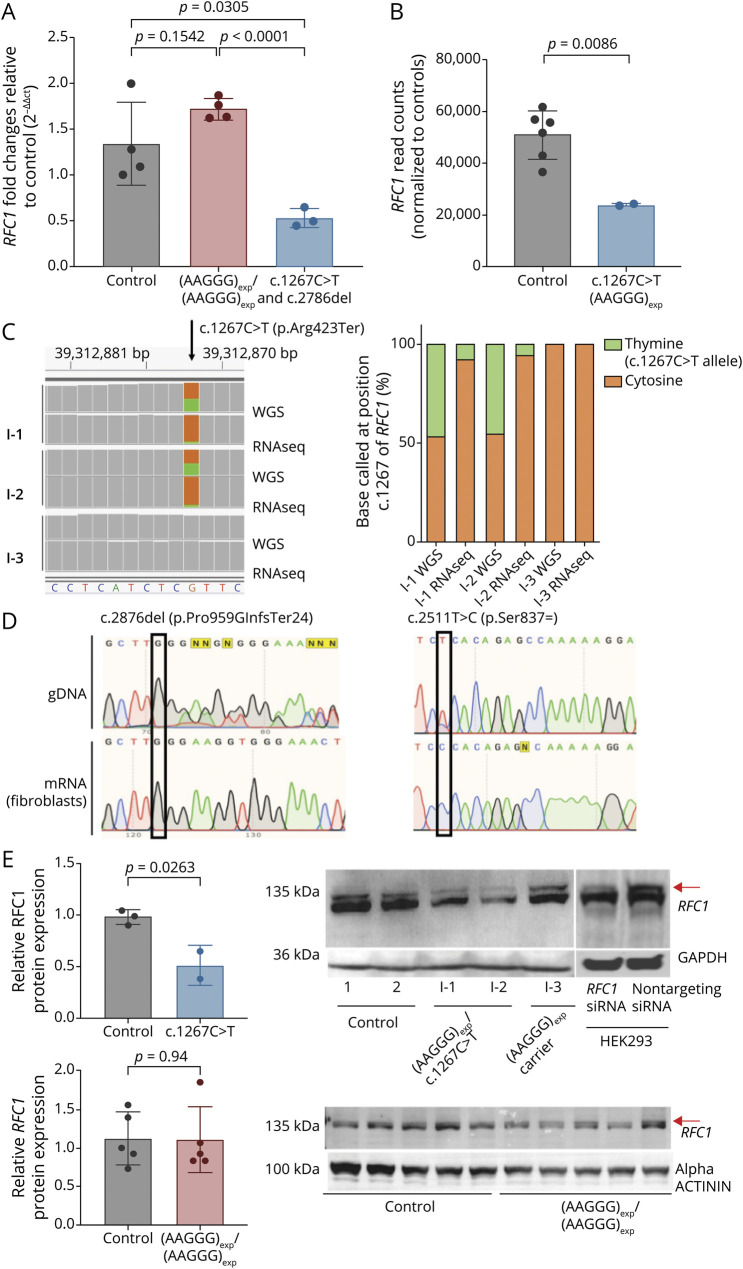
Nonsense-Mediated mRNA Decay and Haploinsufficiency of *RFC1* (A) Real-Time qPCR showed a 40% reduction of *RFC1* mRNA in 2 CANVAS cases carrying c.1267C> T/(AAGGG)_exp_ (n = 2) or c.2876del/(AAGGG)_exp_ (n = 1) mutations compared with healthy controls (n = 4, *p* value = 0.0305) and with biallelic (AAGGG)exp/(AAGGG)exp CANVAS cases (n = 4, *p* value <0.0001). No significant reduction was seen in control vs biallelic CANVAS cases (*p* = 0.154) (B) RNAseq confirmed a significant reduction in *RFC1* transcript level in patients I-1 and I-2 compared with healthy controls (n = 6, *p* = 0.0086). (C) Binary Alignment Map (BAM) data from RNAseq and WGS performed in individuals from family 1 showed in I-1 and I-2 a lower number of reads containing the c.1267C>T mutation (green) compared reads mapping to the transcript derived from the second (brown), as opposed to an equal representation of the 2 alleles on WGS, supporting the presence of nonsense-mediated Decay (NMD) of the c.1267C>T mutant transcript. Schematic representation of the percentage of cytosine and thymine called at c.1267 of *RFC1* in WGS and RNAseq for family 1. (D) Case II RFC1 gDNA and mRNA sequencing. The top left electropherogram show the presence on gDNA of the c.2876del frameshift variant, which is not evident on mRNA sequencing (bottom left) due to nonsense-mediated decay. Conversely, the cytosine allele at base 2,511, which is heterozygously expressed in gDNA and is part of the (AAGGG)_n_ expansion–containing haplotype, seems as “homozygous” in the mRNA sequencing due to nonsense-mediated decay of the second allele, which contains the c.2876del truncating variant. Together, the findings support the location in trans of (AAGG)_n_ repeat expansion and c.2876del variant in this case. (E) Western blotting revealed a 50% reduction of full-length *RFC1* protein expression (red arrow) in CANVAS patients (I-1 and I-2) vs healthy control (control) and their unaffected sibling (I-3) (*p* = 0.0263) along with a reduction of RFC1 in HEK293 cells on siRFC1 transfection. Nontargeting siRNA-transfected cells were used as a control. *RFC1* densitometric values were normalized to GAPDH. No significant reduction in RFC1 protein expression was seen between biallelic (AAGGG)exp/(AAGGG)exp CANVAS patient–derived fibroblasts (n = 5) and healthy control–derived fibroblasts (n = 5) (*p* = 0.94). Abbreviations: CANVAS = cerebellar ataxia, neuropathy, and vestibular areflexia syndrome; RFC1 = replication factor complex subunit 1.

*RFC1* testing was performed and showed the presence of a heterozygous (AAGGG)_n_ repeat expansion in *RFC1*. WES was performed and identified a second c.2876del (p.Pro959GlnfsTer24) truncating variant in *RFC1*. Direct segregation of variants was not possible due to the lack of family members. However, mRNA sequencing studies were performed and provided evidence that (AAGGG)_n_ repeat expansion and c.2876del (p.Pro959GlnfsTer24) variant reside on separate alleles (see paragraph RFC1 expression studies and Figure 2D).

#### Family 3

Case III is a 41 year-old woman who reports progressive imbalance and walking difficulties, particularly in the darkness, since the age of 30 years. Notably, she has always felt clumsy and was unathletic in school. Chronic cough was reported since young age. Oscillopsia and impaired hand dexterity were also later noted, while dysarthria was evident since the age of 40 years. The disease progressed fairly rapidly, and she has been using a walking aid since the age of 38 years and a wheelchair since the age of 40 years. An examination at the age of 41 years showed gaze-evoked and downbeat nystagmus and saccadic pursuit. Vibratory sensation and proprioception were reduced in the toes, while pinprick sensation was reduced to the knees in the lower limbs and the wrists in the upper limbs. Muscle tone and power were normal throughout, while reflexes were increased in the 4 limbs.

Bilateral vestibular impairment was documented by the age of 30 years, while recent investigations showed the association of cerebellar atrophy and sensory neuronopathy.

*RFC1* testing was performed and showed the presence of a heterozygous (AAGGG)_n_ repeat expansion in *RFC1*. WES was performed and identified a second c.1739_1740del (p.Lys580SerfsTer9) truncating variant in *RFC1*. Parental testing was subsequently performed that determined the presence of the (AAGGG)_n_ repeat expansion in this individual's mother and the c.1739_1740del (p.Lys580SerfsTer9) truncating variant in this individual's father, confirming that the variant and the repeat expansion are *in trans* configuration.

#### Family 4

Case IV-1 reports distal sensory loss, itch, and burning pain in his mid-40s, which progressed proximally to involve his trunk and face and which were followed shortly after by imbalance, especially with eyes closed, falls, slurred speech, blurred vision in lateral gaze, and orthostatic hypotension. A dry cough was reported since the age of 20 years. An examination at the age of 47 years showed normal gait but difficulties at tandem walking. There were gaze-evoked nystagmus, saccadic pursuit, abnormal VVOR, and mild dysarthria. Reflexes were brisk, but the motor compartment was otherwise intact. Sensation to pinprick was reduced in the limbs and trunk, vibration was reduced to the knees while proprioception was normal. Investigations were in keeping with full-blown CANVAS, documenting the presence of vestibular areflexia, sensory neuropathy, and cerebellar atrophy.

*RFC1* testing was performed and showed the presence of a heterozygous (AAGGG)_n_ repeat expansion in *RFC1*. WES was performed and identified a second c.2191del (p.Gly731GlufsTer6) truncating variant in *RFC1*. This individual was described to have an older sibling (IV-2) of 54 years diagnosed with ataxia. He is currently wheelchair bound and requires feeding tube due to severe dysphagia. *RFC1* analysis in this older sibling identified the presence of the (AAGGG)_n_ repeat expansion and the c.2191del (p.Gly731GlufsTer6) truncating variant. Parental analysis determined the presence of the c.2191del (p.Gly731GlufsTer6) variant in the father and the (AAGGG)_n_ repeat expansion in the mother, confirming that the variant and the repeat expansion are in trans configuration.

#### Family 5

Case V reported the onset of imbalance at the age of 26 years, particularly at night, which progressed over the years and was associated with chronic cough and mild dysarthria. An examination at the age of 33 years showed a broad-based gait and positive Romberg. There was downbeat nystagmus, exaggerated by lateral gaze, saccadic pursuit, and positive head impulse test. Reflexes were brisk, and notably, tone was increased in the lower limbs with sustained ankle clonus. However, power was normal and Babinski negative. Superficial sensation was reduced with distal-to-proximal gradient, and proprioception was reduced at the toes. There was mild finger-nose and heel-shin ataxia.

Investigations showed the presence of a pure sensory neuropathy with normal motor nerve conduction studies, along with moderate diffuse cerebellar and vermian volume loss at brain MRI and abnormal vestibular testing.

*RFC1* testing was performed and showed the presence of a heterozygous (AAGGG)_n_ repeat expansion in *RFC1*. WES was performed and identified a second c.1267C>T (p.Arg423Ter) truncating variant in *RFC1*. This variant is the same as that detected in family 1. Analysis of this individual's father demonstrated that he carries the c.1267C>T (p.Arg423Ter) truncating variant and does not carry the (AAGGG)_n_ repeat expansion. The mother of this individual was not available for analysis. This result supports that the variant and the repeat expansion are in trans configuration.

### *RFC1* Expression Studies

To determine whether the observed truncating variants would lead to nonsense-mediated mRNA decay and haploinsufficiency of *RFC1*, we evaluated both the RNA and protein expression of the gene (Figure 2, A-E). RT-qPCR was performed on RNA extracted from the fibroblasts of affected I-1, I-2, and II individuals and showed a reduced level of *RFC1* mRNA compared with their unaffected sibling I-3, biallelic AAGGG expansion carriers, and controls (Figure 2A). RNAseq from I-1 and I-2 confirmed the presence of an ∼50% decrease in *RFC1* transcript level (Figure 2B). Notably, the reduced expression was due to the lower number of reads containing the c.1267C>T variant, which accounted for less than 10% of the total number of reads in the region, as opposed to an equal representation of the 2 alleles on WGS (Figure 2C). Touchdown PCR followed by Sanger sequencing also showed nonsense-mediated decay of the allele containing the c.2876del truncating variant in case II. Notably, the (p.Ser837 = ) (rs2066782) synonymous variant, which is part of the haplotype containing the (AAGGG)_n_ expansion, was shown as heterozygous on gDNA from the patient but as “homozygous” on cDNA, suggesting that the c.2876del variant and (AAGGG)_n_ expansion reside on separate alleles (Figure 2D). Together, the findings suggest that the transcript containing truncating variants in RFC1 undergo nonsense-mediated decay leading to RNA degradation. Accordingly, immunoblotting showed a concordant reduction of the 140 KD full-length RFC1 protein in fibroblasts from affected individuals and in siRFC1 HEK293 cell line, compared with biallelic AAGGG expansion carriers and controls, while no truncated isoforms were detected (Figure 2E).

## Discussion

The study reports the first cases of RFC1 CANVAS associated with truncating variants in the *RFC1* gene. Although the molecular consequence of biallelic repeat expansions in *RFC1* remains unsolved, the observation of patients carrying compound heterozygous truncating variant/(AAGGG)_n_ repeat expansion in *RFC1*, resulting in reduction of RFC1 expression, has major implications on future studies on the disease pathogenesis because it supports the existence of a loss-of-function mechanism underlying this condition.

Efficient repair of oxidative DNA damage is an essential function for neurons because they do not replicate and have limited protein turnover.^33^ Therefore, even a subtle impairment of the replication factor complex machinery may be detrimental to this cell type. Indeed, complete loss of RFC1 is not compatible with life in cellular and animal models, and the gene is highly intolerant to haploinsufficiency based on control database data.^27^ Further investigations in disease-relevant tissues are warranted to test this hypothesis.

Patients carrying a completely nonfunctional allele together with a second heterozygous (AAGGG)_n_ expanded allele seemed to show a relatively severe phenotype with dysautonomia and an earlier need for walking aids compared with most patients carrying biallelic (AAGGG)_n_ repeat expansions (Table 1).^6-8^ Notably, 2 cases (III and IV-2) were wheelchair bound by the age of 50 years, and 1 case (IV-2) required tube feeding due to severe dysphagia. Individual V had the earliest onset of the disease, in her mid-20s, and a severe phenotype with clinical involvement of upper motor neurons leading to an increased tone. Brisk reflexes are common in RFC1 disease; however, additional motor features and pyramidal signs are more rarely identified. The observation of a spastic-ataxic syndrome in case V confirms the possibility of clinically manifest upper motor neuron involvement in severe RFC1 disease.

**Table 1:**
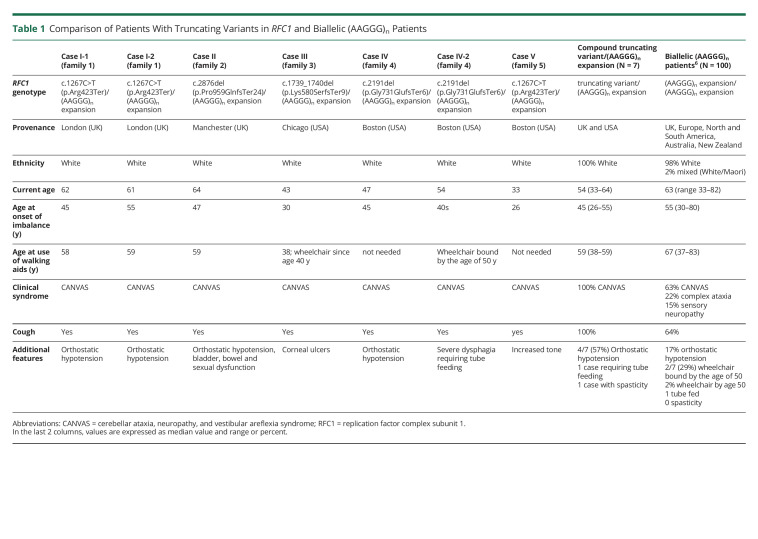
Comparison of Patients With Truncating Variants in *RFC1* and Biallelic (AAGGG)_n_ Patients

Arguably, the identification of truncating variants in *RFC1* leading to a relatively severe phenotype also supports a loss-of-function model of RFC1 disease, where no or very low production of RFC1 protein from 1 allele has more severe functional and clinical consequences compared with biallelic expansions. It is possible that the severity of the disease and clinical phenotype in patients carrying 1 truncating variant was influenced by the expansion size on the second allele. Unfortunately, there was not enough DNA from most cases to perform Southern blotting and test this hypothesis.

Other recessively inherited conditions have previously been associated with compound heterozygous pathogenic variants/repeat expansion genotypes. For example, approximately 1%–4% of individuals with Friedreich ataxia have an abnormally expanded GAA repeat in the disease-causing range on 1 frataxin allele and another intragenic pathogenic variant on the other allele.^34,35^ More recently, noncoding GCA and GCC repeat expansion in compound heterozygous state with a second missense or truncating variant were identified as causative of 2 other rare inherited conditions, glutaminase deficiency^36^ and Baratela-Scott syndrome,^37^ respectively, leading to a clinical phenotype indistinguishable from patients with biallelic pathogenic variants. Of note, loss of function of the repeat-containing genes has been established for all these conditions.

*RFC1* seems intolerant to loss of function, as demonstrated by the presence of only 11 *RFC1* truncating variants of 251,000 alleles present on gnomAD v2.1.1 (allele frequency = 0.00002) with an observed/expected ratio lower than 0.35 (o/e = 0.18, 90% CI = 0.12–0.3) and a very high probability of being loss-of-function intolerant (pLI = 0.97).^27^ Furthermore, there are only 18 *RFC1* truncating variants of 150,000 alleles from individuals enrolled in the Genomics England WGS sequencing project, without (AAGGG)_n_ expansion on the second allele.^38,39^ Biallelic truncating variants are absent from public control databased or Genomics England WGS because they would not be compatible with life.^38,39^ Nonetheless, parents of the affected patients in this study, carrying a truncating variant in *RFC1* but no AAGGG expansion on the second allele, did not show a phenotype despite their old age, suggesting that haploinsufficiency of RFC1, albeit rare in the general population, is tolerated. In fact, a pathogenic mechanism due to hemizygous expression of the repeat-containing allele, which is not sufficient to cause disease when coexpressed with normal protein, cannot be excluded. In this scenario, it could be hypothesized that the only available transcript originating from the repeat-containing allele may exert a similar detrimental effect to biallelic expansions, possibly through gain of function of toxic RNA or repeat peptides. It should be noted that the c.1267C>T (p.Arg423Ter) variant that was detected in 2 independent families in our cohort is present in the gnomAD population database in 2 individuals, one of East Asian and the other of European (non-Finnish) ancestry. The c.1267C>T change does occur at a CG dinucleotide, which may explain its susceptibility to being mutated, and it may represent a more common pathogenic variant in CANVAS.

In 7 families with typical CANVAS and 1 expanded (AAGGG) allele, a definite cause was not identified, suggesting the possible presence of genetic heterogeneity underlying CANVAS phenotype. Our report expands the genotype spectrum of RFC1 disease and has direct diagnostic implications. Indeed, albeit rare in our cohort (7 cases vs more than 650 individuals carrying biallelic *RFC1* expansions, ∼1%), the finding of truncating variants associated with CANVAS indicate that full *RFC1* sequencing is recommended in cases affected by typical CANVAS and carrying monoallelic (AAGGG)_n_ expansions.

## Supplementary Material

Download Supplementary Video 1

## Glossary

CANVAS: cerebellar ataxia, neuropathy, and vestibular areflexia syndrome
ESP: Exome Sequencing Project
NHLBI: National Heart, Lung, and Blood Institute
RFC1: replication factor complex subunit 1
VVOR: visually enhanced vestibulo-ocular reflex
WES: whole-exome sequencing
WGS: whole-genome sequencing
